# Acute Febrile Illness and Complications Due to Murine Typhus, Texas, USA^1^,^2^

**DOI:** 10.3201/eid2308.161861

**Published:** 2017-08

**Authors:** Zeeshan Afzal, Sunand Kallumadanda, Feng Wang, Vagish Hemmige, Daniel Musher

**Affiliations:** University of Texas Rio Grande Valley, McAllen, Texas, USA (Z. Afzal, S. Kallumadanda, F. Wang);; Baylor College of Medicine, Houston, Texas, USA (V. Hemmige, D. Musher);; M.E. DeBakey VA Medical Center, Houston (D. Musher)

**Keywords:** acute febrile disease, nonspecific symptoms, rickettsial diseases, rickettsia, murine typhus, vector-borne infections, zoonoses, Texas, Rickettsia typhi

## Abstract

Murine typhus occurs relatively commonly in southern Texas, as well as in California. We reviewed records of 90 adults and children in whom murine typhus was diagnosed during a 3-year period in 2 hospitals in southern Texas, USA. Most patients lacked notable comorbidities; all were immunocompetent. Initial signs and symptoms included fever (99%), malaise (82%), headache (77%), fatigue (70%), myalgias (68%), and rash (39%). Complications, often severe, in 28% of patients included bronchiolitis, pneumonia, meningitis, septic shock, cholecystitis, pancreatitis, myositis, and rhabdomyolysis; the last 3 are previously unreported in murine typhus. Low serum albumin and elevated procalcitonin, consistent with bacterial sepsis, were observed in >70% of cases. Rash was more common in children; thrombocytopenia, hyponatremia, elevated hepatic transaminases, and complications were more frequent in adults. Murine typhus should be considered as a diagnostic possibility in cases of acute febrile illness in southern and even in more northern US states.

Murine (endemic) typhus is most frequently recognized as an acute febrile illness, although the clinical manifestations of this infection cover the full spectrum of disease, from asymptomatic infection to fulminant disease and death (*1*–*5*). The causative organism, *Rickettsia typhi*, is a small, gram-negative, obligate intracellular bacterium. Worldwide, especially in tropical and subtropical areas, the roof rat (*Rattus rattus*) and Norway rat (*Rattus norvegicus*) have been the principal reservoirs, with the rat flea, *Xenopsylla cheopis*, as the principal vector (*2**,**6*).

In the United States, individual murine typhus cases and outbreaks have been reported from suburban areas; in these instances, opossums (*Didelphis virginiana*) and *Rattus* spp. have been implicated as the reservoirs, with the cat flea, *Ctenocephalides felis*, as the principal vector (*2*). The incidence of murine typhus declined sharply in the United States after the institution of DDT for control of rat fleas in 1945 (*5*), but it now appears to be on the rise, especially in southern Texas and California (*2*,*7*,*8*). We describe 90 murine typhus infections that were diagnosed in 2 hospitals in Hidalgo County, Texas, USA, during a 3-year period, with a comparison of clinical manifestations in adults and children.

## Methods

### Patient Identification

We searched microbiology laboratory records at McAllen Medical Center (McAllen, TX, USA) and Edinburg Regional Medical Center (Edinburg, TX, USA) for July 1, 2013–June 30, 2016, to identify patients who had typhus group rickettsiae IgM or IgG at a titer >1:128 (*9*) (Focus Diagnostics Rickettsia, Indirect Immunofluorescence Antibody for IgM and IgG; Labcorp, Houston, TX, USA). A total of 101 patients were identified; all had been hospitalized. We carefully reviewed their medical records to be certain that their clinical syndrome was suggestive of murine typhus.

Eleven of the 101 patients were excluded: 5 had incomplete medical records sufficient to make a clinical diagnosis and 6 had another condition that might have caused a fever (otitis media, active hepatitis C, Epstein-Barr virus infection, untreated HIV infection, lymphoma, or tuberculosis). IgG titers had been measured in every case. Of the remaining 90 patients, 41 (45.6%) had been evaluated by an infectious disease consultant, who either concurred with or suggested the diagnosis of murine typhus in every case.

### Case Definitions

For case identification, we used the Flea-borne Typhus Epi Case Criteria 2016 Case Definition/Case Classification of the Texas State Department of Health (*9*). We also used the case classification scheme described by the Centers for Disease Control and Prevention for spotted fever rickettsiosis 2010 to categorize our patients with suspected murine typhus, probable murine typhus, or confirmed murine typhus (*10*). Patients with suspected murine typhus were those with laboratory evidence of past or present infection but without a clinically compatible illness. Patients with probable murine typhus were those in whom the clinical disease was compatible with murine typhus (meets clinical evidence criteria) and with supportive laboratory results. Patients with confirmed murine typhus had a clinically compatible illness and laboratory confirmation. Illnesses that are clinically compatible with murine typhus are characterized by acute onset of fever and >1: headache, myalgia, anorexia, nausea/vomiting, thrombocytopenia, or elevated hepatic aminotransferases. Supportive laboratory evidence is defined by indirect immunofluorescence assay (IgM or IgG) serologic titer >1:128. Laboratory-confirmed cases are required to have serologic evidence of a 4-fold increase in IgG-specific antibody titer reactive with typhus group rickettsiae antigen by indirect immunofluorescence assay in paired serum specimens (1 taken in the first week of illness and a second 2–4 weeks later).

### Data Gathering

We reviewed electronic medical records to obtain data on demography and epidemiology; comorbid conditions; symptoms and their duration before hospitalization; physical findings; laboratory results (hematology, chemistry, and microbiology); imaging; requirements for intensive care; hospital course; antimicrobial drug treatment; response to treatment; and duration of hospital stay. This study was approved by the institutional review board of the University of Texas Rio Grande Valley Medical School.

### Data Analysis

Basic descriptive statistics were calculated, and comparisons between the proportion of adult and pediatric patients with different signs and symptoms of murine typhus were calculated with a χ^2^ or Fisher exact test, as appropriate. All analyses were performed in Stata 12 (Statacorp LLC, College Station, TX, USA).

## Results

On the basis of the definitions we described, 90 patients were given a diagnosis of murine typhus. One patient had a clinical syndrome consistent with murine typhus but the presence of fever was not documented; this illness was characterized as suspected murine typhus. Eighty-six patients met criteria for probable murine typhus, and 3 had confirmed murine typhus. Overall, the initial IgM titer was >1:128 in 86 (96%) cases, and the IgG titer was >1:128 in 63 (70%) cases. Five patients had a second blood sample submitted for typhus group rickettsiae IgG; 3 of these exhibited a 4-fold increase in IgG titer (rising from <1:64 to 1:512) and were determined to be confirmed murine typhus. One patient had a 2-fold increase in IgG titer; in the other patient, IgG titer remained unchanged but IgM titer doubled from 1:128 to 1:256, thus meeting the criteria for probable murine typhus.

Of the 90 patients, 36 (40%) were <18 years of age, and 45 (50%) were female. All patients included in the final analysis had a clinical syndrome suggestive of murine typhus (Table 1), including fever in 99%, malaise in 82%, headache in 77%, fatigue in 70%, myalgias in 68%, and rash in 39%. Other causes of the syndrome were sought by bacteriologic and serologic studies in a varying proportion of these cases; no other cause was found. 

**Table 1:** Characteristics of 90 patients with murine typhus, Hidalgo County, Texas, USA, July 1, 2013–June 30, 2016*

Characteristic	No. (%) patients			
	Age ≥18 y	Age <18 y	Total	p value
Symptoms				
Subjective fever/chills	53/54 (98)	36/36 (100)	89/90 (99)	1
Malaise	21/24 (88)	15/20 (75)	36/44 (82)	0.44
Headache	37/46 (80)	23/32 (72)	60/78 (77)	0.38
Fatigue	22/29 (76)	10/17 (59)	32/46 (70)	0.22
Myalgias	29/39 (74)	13/23 (57)	42/62 (68)	0.15
Nausea and/or vomiting	31/52 (60)	21/35 (60)	52/87 (60)	0.97
Cough	21/52 (40)	18/36 (50)	39/88 (44)	0.37
Abdominal pain	17/47(36)	10/33 (30)	27/80 (34)	0.58
Arthralgia	9/34 (26)	4/24 (17)	13/58 (22)	0.53
Diarrhea	9/50 (18)	1/35 (3)	10/85 (12)	0.04
Confusion	6/42 (14)	2/30 (7)	8/72 (11)	0.45
Signs				
Temperature ≥100.4°F	48/54 (89)	34/36 (94)	82/90 (91)	0.47
Rash	16/51 (31)	18/36 (50)	34/87 (39)	0.12
Enlarged liver	1/44 (2)	1/32 (3)	2/76 (3)	1
Enlarged spleen	0/44 (0)	1/32 (3)	1/76 (1)	0.42
Laboratory findings				
Leukocytes <6,000 cells/mm^3^	10/54 (19)	11/36 (31)	21/90 (23)	0.19
Leukocytes <3,000 cells/mm^3^	0	0	0	1
Platelets <120,000/mm^3^	37/54 (69)	12/36 (33)	49/90 (54)	<0.01
Bilirubin ≥1.5 mg/dL	14/54 (26)	3/36 (8)	17/90 (19)	0.05
AST >50 IU/L	51/54 (94)	25/36 (69)	76/90 (84)	<0.01
ALT >50 IU/L	45/54 (83)	22/36 (61)	67/90 (74)	0.02
Serum sodium ≤133 mmol/L	34/54 (63)	6/36 (17)	40/90 (44)	<0.01
Serum albumin <3.5 g/dL	46/54 (85)	27/36 (75)	73/90 (81)	0.23
Serum procalcitonin >0.5 ng/mL	9/13 (69)	1/1 (100)	10/14 (71)	1
IgM ≥1:128	50/54 (93)	36/36 (100)	86/90 (96)	0.15
IgG ≥1:128	41/54 (76)	22/36 (61)	63/90 (70)	0.13
Complications	18/54 (33)	7/36 (19)	25/90 (28)	0.15

*ALT, alanine aminotransferase; AST, aspartate aminotransferase.

Complications of murine typhus occurred in 25 (28%) cases. Among documented complications were bronchiolitis in 2 cases, pneumonia in 8, pancreatitis in 3, cholecystitis in 1, myositis (creatine phosphokinase >1,500 U/L) in 1, rhabdomyolysis (creatine phosphokinase >4,000 U/L) in 2, meningitis in 2, sepsis with acute kidney injury in 1, and septic shock in 4. In 1 patient, pneumonia and septic shock occurred together. There were no deaths.

Laboratory findings consistent with murine typhus included leukocytes <6,000 cells/mm^3^ in 21 (23%) patients, platelets <120,000/mm^3^ in 49 (54%), aspartate aminotransferase >50 IU/L in 76 (84%), alanine aminotransferase >50 IU/L in 67 (74%), and serum sodium <133 mmol/L in 40 (44%). In no case was the leukocyte count as low as 3,000 cells/mm^3^. Serum albumin was <3.5 g/dL in 73 (81%) of 90 cases, and serum procalcitonin was >0.5 ng/mL in 10 (71%) of 14 cases, both findings consistent with bacterial sepsis. Newly recognized complications of murine typhus in this case series included pancreatitis, myositis, and rhabdomyolysis. Thirteen patients (14%) required care in the intensive care unit.

We found notable differences between adult and pediatric patients (Table 1) in the frequency of diarrhea, platelet count <120,000/mm^3^, serum sodium ≤133 mmol/L, and aspartate aminotransferase and alanine aminotransferase >50 IU/L, all of which were more common in adults. Complications occurred in 18 (33%) of 54 adults and 7 (19%) of 36 children (p = 0.15).

The median duration from onset of illness to hospitalization was 7 days (range 1–21 days). Many patients saw a physician before coming to the hospital. We determined that the median time between the initial clinical encounter and institution of effective antimicrobial drug therapy was 5 days (range 0–15 days). A wide array of antimicrobial drugs was prescribed. Eight patients never received an antimicrobial drug appropriate to treat murine typhus, but the disease resolved spontaneously.

## Discussion

Murine typhus causes a syndrome characterized by nonspecific manifestations, including fever, headache, myalgias, malaise, nausea, and vomiting in more than half of cases. This syndrome has previously been described throughout the world, including the southwestern United States (*1*,*4*,*5*,*11*–*13*) and California (*2*,*14*), as well as Europe (*3*,*15*–*17*). 

Twenty-three of the 90 patients in this study were first seen in a primary care physician’s office. Diagnoses made at the time of that initial encounter, whether in children or adults, included viral syndrome, influenza, and streptococcal pharyngitis; murine typhus was not considered in any of them. Once patients were hospitalized, this nonspecific clinical syndrome, together with characteristic laboratory abnormalities (low leukocyte and platelet counts and elevated levels of liver enzymes) and knowledge of the occurrence of murine typhus in this area, enabled the correct diagnosis to be considered. Serum samples were submitted for supportive laboratory testing, and appropriate therapy was instituted promptly, in most cases before results of serologic tests were known. It is still worth noting that, for 8 patients, the diagnosis of murine typhus was never made during their illness, but only when serologic results were returned. Illness resolved spontaneously in these 8 patients. 

Serum procalcitonin, not previously reported in murine typhus, was elevated in 10 (71.4%) of the 14 cases in which it was studied; this test might help distinguish murine typhus from a viral syndrome in an endemic area. The low serum albumin is also consistent with bacterial sepsis (*18*).

Although the usual symptoms were those of a nonspecific illness suggestive of influenza, another viral syndrome, or streptococcal pharyngitis, many of our patients were, at some point, quite ill. Thirteen (14%) of 90 were hospitalized in an intensive care unit. Six met criteria for sepsis; serum albumin fell to <3.5 g/dL, consistent with serious bacterial infection, in 73 (81%) of the 90 patients. Complications of murine typhus, defined as symptoms, signs, or both beyond nonspecific ones, were seen in 25 (28%) patients and appeared more frequently in adults than in children (Table 2). The most common complications were pulmonary complications, such as bronchiolitis and pneumonia; aseptic meningitis; and sepsis with shock. These complications have been described previously, either in case reports or in case series (*1*,*2*,*12*,*16*,*17*,*19*). Only 2 previous studies have specifically tabulated complications (*3*,*20*), with the frequency of complications in 1 prospective series being similar to that in this study (*20*). 

**Table 2:** Complications in 90 patients with murine typhus, Hidalgo County, Texas, USA, July 1, 2013–June 30, 2016

Complication	Age ≥18 y, n = 54	Age <18 y, n = 36	Total, n = 90
Bronchiolitis	0	2	2
Pneumonia	7	1	8
Pancreatitis	3	0	3
Cholecystitis	0	1	1
Myositis	0	1	1
Rhabdomyolysis	0	2	2
Meningitis	2	0	2
Septic shock	4	0	4
Sepsis with acute kidney injury	1	0	1
Pneumonia plus septic shock	1	0	1
Total	18	7	25

No previous investigation has reported on serum procalcitonin, which was elevated in 10 (71.4%) of 14 patients in the study in which it was measured. Newly described complications in this series included pancreatitis, myositis, and rhabdomyolysis. Unusual cutaneous manifestations (*21*), iritis (*20*), lymphadenopathy (*20*), splenic rupture (*22*,*23*), disseminated intravascular coagulation (*3*,*12*), cholecystitis (*24*), and endocarditis (*25*,*26*) have previously been reported; except for 1 patient with cholecystitis, we had no examples of these other unusual complications in our series.

Earlier series of cases have not compared findings in adults and children. Differences between children and adults in this study included greater frequency of rash in children, whereas statistically significant differences were greater incidence of diarrhea, thrombocytopenia, hyponatremia, and elevated hepatic aminotransferases in adults. Complicated illness was more common in older than in younger subjects, as has been suggested in separate reports from a group of investigators in Greece (*16*,*17*).

Murine typhus has been increasingly recognized in Texas (*8*). Two counties in Texas have reported >50 cases of murine typhus per year (*1*,*11*), and in 1 of these, a high prevalence of antibodies to *R. typhi* has been described in healthy children (*13*). To our knowledge, such a study has not been reported in adults in southern Texas, the area of greatest endemicity in the United States. Our finding of 90 recognized cases of murine typhus in 2 hospitals in a 3-year period attests to the frequency of murine typhus in the southwestern United States.

Rats and opossums that coexist in urban and suburban settings appear to be reservoirs for the organism. The vector has traditionally been thought to be *Xenopsylla cheopis*, the rat flea (*6*). However, recent studies (*27*,*28*) have shown that the cat flea, *Ctenocephalides felis*, which also infests opossums, plays a role in transmission. Blanton et al. (*27*) found rickettsial DNA in pools of fleas obtained from opossums in Galveston, Texas, and documented the emergence of antibodies to *Rickettsia* sp. in these animals. A major outbreak of murine typhus in Austin, Texas, was traced to contact with opossums (*11*). Because *R. felis* may also be present in fleas living on these same reservoirs (*29*,*30*) and the clinical spectrum of infection due to this organism remains undefined, it is possible that some cases attributed to *R. typhi* and identified as murine typhus may actually be due to *R. felis*.

The natural habitat of the American opossum includes northeastern and northwestern states, suggesting the possibility that murine typhus may not be confined to areas that are currently recognized as endemic, such as southern Texas or California. In Virginia and the Carolinas, illnesses of patients with fever, rash, and nonspecific symptoms are regularly diagnosed as Rocky Mountain spotted fever and treated with doxycycline; however, it is possible that some of these infections may be due to *R. typhi*. PCR (*24*,*31*,*32*) can distinguish among these organisms, but it is not readily available and has uncertain clinical value. *R. felis* may also cause human infection and requires PCR to distinguish it from *R. typhi* (*29*,*30*,*33*). In fact, as suggested by Eremeeva et al. (*29*), murine typhus as we now know it may be a single clinical syndrome that can be caused by either *R. typhi* or *R. felis.*

The principal limitation of our study is that diagnoses were suspected clinically but were supported by detection of serum antibodies reactive with typhus group rickettsial antigen. Cross-reacting IgM may also appear after infection with other organisms in the typhus group (*R. prowazecki*) and the spotted fever group (*R. rickettsii*, the cause of Rocky Mountain spotted fever), as well as *R. felis*; we cannot exclude the possibility that some of our cases may have been caused by these organisms. Other cases of *R. typhi* infection may have been missed because physicians suspected the disease very early and instituted treatment with doxycycline. The principal strengths of this study are that the medical records were reviewed in every case by the principal investigator and that many of the patients had been seen by an infectious disease consultant who concurred with the final diagnosis, usually before serologic results were available.

In conclusion, murine typhus is a multifaceted disease that is common in southern Texas and California and that might be identified more frequently in other areas of the United States if the diagnosis were sought. A major description of this disease, especially with newly proposed case definitions, has not appeared in an American journal for 15 years; pediatric and adult cases have not been compared, and the range of complications in the United States has not been described recently. The population of the American opossum has increased in northern states. In Texas and California, their fleas are known to carry *R. typhi* (*27*,*34*) but, to our knowledge, a search for *Rickettsia* spp. in opossums has not been conducted in other parts of the United States. We suggest that, in cases of acute febrile conditions, the diagnosis of murine typhus needs to be considered elsewhere in the southern United States and, perhaps, farther north as well. Because of the high prevalence of murine typhus in areas of recognized endemicity, medical centers might consider on-site testing for antibodies to typhus group rickettsiae to support a clinical decision and early treatment. A reactive serology might then trigger a reflex request to obtain convalescent titers to provide confirmatory data.
